# Women Who Sleep More Have Reduced Risk of Peptic Ulcer Disease; Korean National Health and Nutrition Examination Survey (2008–2009)

**DOI:** 10.1038/srep36925

**Published:** 2016-11-10

**Authors:** Sun-Hye Ko, Myong Ki Baeg, Seung Yeon Ko, Kyung-Do Han

**Affiliations:** 1 Department of Internal Medicine, Asan Medical Center, University of Ulsan College of Medicine, Seoul, South Korea; 2 Department of Internal Medicine, International St. Mary’s Hospital, College of Medicine, Catholic Kwandong University, Incheon, South Korea; 3 Institute for Translational and Clinical Research, Catholic Kwandong University, Incheon, South Korea; 4 Department of Surgery, Incheon St. Mary’s Hospital, College of Medicine, The Catholic University of Korea, Incheon, South Korea; 5 Department of Preventive Medicine, The Catholic University of Korea, Seoul, South Korea

## Abstract

Sleep is integral to life and sleep duration is important in sleep quality, physical, and psychological health. Disturbances in sleep duration have been associated with increased risk of metabolic disorders, hypertension, and overall mortality. Sleep disturbance has also been linked with various gastrointestinal disorders. However, the association between sleep and peptic ulcer disease (PUD) has not been evaluated. We investigated the association between sleep duration and PUD. Subjects were included from the fifth Korean National Health and Nutrition Examination Survey conducted from 2008–2009. Individuals with PUD were defined as those with a physician diagnosis of PUD. Daily sleep duration was established by asking participants the amount of time that they slept per day. Multiple logistic regression models were used to evaluate the association of PUD and sleep duration. This study included 14,290 participants (8,209 women). The prevalence of PUD was 5.7% and was higher in men (6.8%) than in women (4.9%). Women who slept ≥9 hours were significantly less likely to have PUD compared to women who slept 7 hours. In men, longer sleep duration (≥9 hours) had a tendency toward PUD prevention. Our results suggest that longer sleep duration may play a protective role for PUD development.

Sleep is an essential part of human life and sleep duration is an important factor in sleep quality, physical, and psychological health^1^,^2^,^3^,^4^,^5^. Previous studies have reported that short or long durations of sleep are associated with an increased risk of obesity, hypertension, type 2 diabetes, metabolic syndrome, and overall mortality^1^,^3^,^4^,^6^,^7^,^8^. Sleep disturbance has also been linked to gastrointestinal diseases^9^,^10^. During sleep, defensive mechanisms against peptic ulcer disease (PUD) including gastric mucosal blood flow, gastric bicarbonate efflux, and melatonin secretion have been reported to increase while gastric acid secretion is decreased^9^,^11^.

According to a recent systematic analysis for the Global Burden of Disease study, PUD is a common and serious medical problem^12^,^13^. The prevalence of PUD is approximately 4.1%, and about 10% of people develop PUD during their lifetime^13^,^14^. Though *Helicobacter pylori* and nonsteroidal anti-inflammatory drugs are the most important risk factors for PUD, no obvious cause is found in 5–20% of PUD patients^15^. Recent studies suggest that non-organic causes such as psychological stress may play a role in the onset and course of PUD^15^,^16^,^17^.

The association between sleep duration and PUD remains to be fully understood. In this study, we assessed the association between sleep duration and PUD among adults representative of the Korean population.

## Results

### Characteristics of participants

Of the 20,277 KNHANES IV participants, 15,071 were eligible for this study. 14,290 participants (6,081 men and 8,209 women) were included and 781 subjects were excluded due to missing data for variables. The prevalence of PUD in the entire population was 5.7%, and the prevalence of PUD was higher in men (6.8%) than in women (4.9%).

Table 1 shows the clinical characteristics of the study participants by sex. Participants with PUD were older and had a lower education level than participants without PUD for men and women. There were no differences in smoking, drinking and exercise habits between those with and without PUD. However, subjects with PUD indicated that they were under more stress, had suicidal ideation, and more depressive symptoms than those without PUD. The mean sleep duration of participants with PUD was significantly shorter than those without PUD, regardless of sex.

### Characteristics of the study population according to sleep duration

The prevalence of PUD and the characteristics of the study population according to sleep duration are presented in Table 2. The reference group was established as men (28.3%) and women (28.4%) who slept 7 hours per day. Drinking and exercise were significantly associated with sleep duration among men. Current smoking and exercise were statistically significant across the sleep duration categories for women. Participants with sleep durations of <5 hours were more likely to be older, obese and under stress compared with any other sleep group. Subjects who had depressive symptoms, and suicidal ideation exhibited a U-shaped prevalence of PUD with sleep duration, regardless of sex.

### Associations between sleep duration and PUD

The relationship between sleep duration and PUD after adjustment for multiple risk factors can be seen in Table 3. In age and BMI adjusted logistic regression analysis, women who slept ≥9 hours were less likely to have PUD (Odds Ratio (OR) = 0.59, 95% Confidence Interval (CI) = 0.363–0.950) compared to those who slept 7 hours (reference group). After further adjustment for smoking, drinking, exercise, education, income, and spouse, the negative association between PUD and long sleep duration (≥9 hours) persisted in women (OR = 0.60, 95% CI = 0.369–0.978). When stress and depressive symptoms were added to logistic regression models for full adjustment, women with long sleep duration (≥9 hours) had lower PUD than those with 7 hours sleep (OR = 0.59, 95% CI = 0.362–0.964). Long sleep duration (≥9 hours) had a preventive effect tendency for PUD through all models in men.

When suicidal ideation instead of the depressive symptoms was fitted into the fully adjusted model, the findings did not change substantially (data not shown).

## Discussion

In this study, we demonstrated that longer sleep duration is a potential preventive factor for PUD in women. The association between sleep duration and PUD was not significant in men. These associations remained evident even after adjusting for established risk factors, such as old age, smoking, drinking and stress, thus reinforcing our conclusion that longer sleep duration was an independent protective factor for PUD in women. To our knowledge, this is the first population-based study to assess the association between sleep duration and PUD.

Several studies have indicated a relationship between sleep duration and cardiovascular diseases, obesity, hypertension, type 2 diabetes, metabolic syndrome, and overall mortality^1^,^2^,^3^,^4^,^5^,^6^,^8^. However, the association between sleep duration and PUD has not been evaluated. One study reported a positive association between sleep and peptic ulcer bleeding from a nationwide population base-study^18^. In this study, patients with sleep apnea had a 2.4-fold higher risk for incident peptic ulcer bleeding^18^. Hyperactivation of the sympathetic system and increased blood pressure may have caused peptic ulcer bleeding in a manner analogous to stress ulcer formation, which may also have contributed to the increased incidence of PUD in our study^19^,^20^.

PUD is clinically important because it is a common disorder with one in ten Americans developing PUD^14^. The disease adds a substantial burden to patients, health care professionals, and the health care system. The pathogenesis of PUD lies in the imbalance between the gastroduodenal mucosal defense system and gastric acid secretion^21^. Stress and anxiety were accepted as the main causes of PUD in the mid-20th century^22^. Considerations of etiology evolved with the discovery of the relationship between *Helicobacter pylori* and PUD^23^. However, recent studies suggest that psychological stress may play a role in the onset and the course of PUD^15^,^16^,^17^. Our findings suggest that sleep duration may be a novel risk factor for PUD.

The mechanism underlying the relationship between sleep duration and PUD is not clear, but there are some potential explanations. One is increased gastric mucosal blood flow and decreased gastric acid secretion during sleep. Increased gastric mucosal blood flow accelerates ulcer healing and decreased gastric mucosal blood flow has been reported to cause acute gastric mucosal lesions^24^. In addition, gastric acid secretion has been reported to decrease in deeper stages of sleep, especially during rapid eye movement while sleeping^25^. This may be associated with reduction in plasma noradrenaline, gastrin, and histamine levels and increasing gastric mucosal blood flow^26^.

Another mechanism explaining the relationship between sleep and PUD may be related to melatonin. Melatonin has been reported to be a potent stimulant of bicarbonate and may contribute to the healing of gastric lesions. This may be due to inhibition of gastric acid secretion, enhancement of gastric mucosal blood flow, and influence on prostaglandin-dependent pathways^27^,^28^. Melatonin has been demonstrated to have antistress neurohormonal properties and may exert gastrointestinal protection by other mechanisms, including scavenging oxygen radicals and increasing mucosal microcirculation and cell proliferation^9^,^27^,^28^.

Interestingly, we only found a significant association between sleep duration and PUD in women. Consistent with our findings, previous studies have shown sex-dependent associations between sleep duration and hypertension and metabolic syndrome^3^,^29^,^30^,^31^. Although we could not explain the sex-specific effects of sleep duration on PUD at the present time, it is possible that sex-related hormonal influences, sex differences in stress, and reaction to stress may play a role^32^. A recent analysis reported that women are more vulnerable to changes in sleep duration^33^. Another study reported that women were found to secrete melatonin earlier and have a significantly higher melatonin amplitude than men with a similar sleep duration^33^. This may account for women with longer sleep duration having a reduced risk of PUD, where men had a nonsignificant difference. Measuring melatonin levels may have helped support our study results. Further research between sleep duration, melatonin levels and gender may help explain this in the future.

There were some limitations to this study. First, PUD was defined as previous diagnosis by a physician. PUD requires endoscopic confirmation, which suggests that some of the subjects in our study may not have had PUD. However, the incidence of endoscopically confirmed PUD has been reported to be between 2–10% in Korea, which suggests that our study may not have overestimated PUD incidence^34^,^35^,^36^,^37^. Also, as the lifetime screening rate for stomach cancer during the study period was about 65%, we believe that many of our subjects had been diagnosed with PUD by endoscopy^38^. Furthermore, upper gastrointestinal endoscopy is readily available in Korea, costs less than $35 and is offered free of charge in the biennial government or yearly employee health examinations^39^,^40^. As such, we believe that the physician reported diagnosis is credible. Second, we relied on self-reported sleep duration instead of objective measures of sleep duration. However, we considered the self-reports sufficient, as previous studies have shown good agreement between self-reported sleep durations and those obtained through actigraphic monitoring^41^. Third, though age was included in the multivariable analysis, there was a significant age difference between the PUD and non-PUD groups. Older age is associated with aging of the mucosa and increased comorbidities, which may have influenced the outcome^42^. Fourth, PUD risk factors such as *H. pylori* status and drug history including non-steroidal anti-inflammatory drugs or antidepressants were not investigated in KNHANES. Despite these limitations, our study has merit, as KNHANES is representative of the entire Korean population and is large enough to provide adequate power.

## Conclusions

Our results suggest that longer sleep duration (≥9 hours a day) could play a protective role in the development of PUD. Further prospective longitudinal studies are needed to identify the biological mechanisms underlying sleep duration and PUD risk with better control of confounders in different ethnic or age groups. If sleep duration plays a causative role in PUD, increased sleep could help in the prevention of PUD and improve public health.

## Materials and Methods

### Data source and subjects

This study was based on data acquired from the fifth Korean National Health and Nutrition Examination Survey (KNHANES IV) conducted from 2008–2009. The KNHANES is a nationwide, cross-sectional survey used to estimate the general health and nutrition status of the Korean population, conducted by the Korea Centers for Disease Control and Prevention (KCDCP). It consisted of a health interview, physical examination, and nutrition survey. Additional details regarding the design and methods of the survey have been described elsewhere^43^. KNHANES IV used a stratified multistage cluster probability sampling by a rolling sampling method, which was certified as producing representative statistics by the Korean Department of Statistics.

Participants older than 19 years of age were included in this study. Subjects with data missing for variables included in the analysis were excluded. The survey protocol was conducted according to the Declaration of Helsinki and approval for this study was granted by the Institutional Review Board of the Catholic University Medical Center (IRB No. KC15EISI0840). All survey participants signed informed consent forms.

### Measurements

Anthropometric measures of the participants were performed by trained personnel. Waist circumference (WC) was measured at the midpoint between the lowest rib and the iliac crest. Blood pressure (BP) was measured in a seated position using a mercury sphygmomanometer (Baumanometer; Baum, Copiague, NY, USA). Three measurements were made with at least five minutes intervals and the mean value of the second and third measurements were used.

Cigarette smoking was classified as current smokers for participants who had smoked ≥100 cigarettes and currently smoked. For alcohol consumption, a heavy drinker was defined as an individual who consumed ≥30 g alcohol/day. The definition of regular exercise was moderate physical activity ≥30 minutes per day for >5 days per week and/or strenuous physical activity for ≥20 minutes per day for >3 days per week. Education level was categorized into less than high school, high school, and college or higher. Marital status was classified as “spouse, yes” for participants who were married and lived with their spouse and “spouse, no” for those who were unmarried, widowed, divorced, or separated. Household income was measured as quartiles based on per capita household income. Stress level was reported as none, mild, moderate and severe during the routine daily life. When a participant checked ‘moderate’ or ‘severe’, this participant was classified as being in the stressful state. Depressive symptoms were determined based on the question, “Have you felt sadness or despair which affected your daily life for more than 2 weeks over the past year?” Suicidal ideation was defined as thoughts of committing suicide during the past year.

Blood samples were collected in the morning after at least eight hours of fasting. Samples were properly processed, refrigerated immediately, and analyzed within 24 hours at the Central Testing Institute in Seoul, Korea. Fasting plasma glucose, total cholesterol, high-density lipoprotein cholesterol, and triglyceride were measured using the Hitachi Automatic Analyzer 7600 (Hitachi, Tokyo, Japan).

### Definitions

Individuals with PUD were defined as those with a diagnosis of PUD by a physician. Daily sleep duration was established by asking the open-ended question: “How much time do you usually sleep per day?” Diabetes was classified as fasting plasma glucose ≥126 mg/dL, current antidiabetic medication, or a previous diagnosis of type 2 diabetes by a physician. Individuals with hypertension were defined as those with a systolic BP ≥ 140 mmHg, a diastolic BP ≥ 90 mmHg, or self-reported current use of antihypertensive medications.

### Statistical analysis

We used the KNHANES sampling weight variables with stratification, designated by the KCDCP, which were based on the sample design of each survey year. Data are presented as the mean ± standard error (SE) for continuous variables or as proportions (SE) for categorical variables. The means of continuous variables were compared by independent *t*-tests, and the proportion of categorical variables were tested by Pearson’s chi-square tests.

Prevalence of PUD was measured for each sex, and further determined in each sleep duration group by sex. Multiple logistic regression models were used to evaluate the association of PUD with sleep duration. Three models were constructed: in model 1, adjustments were made for age and body mass index; model 2 included additional adjustments for smoking, drinking, exercise, education, income, and spouse; and in model 3, adjustments for stress and depressive symptoms were added. These anayses were repeated after replacing depressive symptoms with suicidal ideation. Results are presented as odds ratios and 95% confidence intervals. Differences were considered to be significant if the p-value was less than 0.05. Statistical analyses were performed using SAS for Windows (version 9.20, SAS Institute, Cary, NC, USA).

## Additional Information

**How to cite this article**: Ko, S.-H. *et al*. Women Who Sleep More Have Reduced Risk of Peptic Ulcer Disease; Korean National Health and Nutrition Examination Survey (2008–2009). *Sci. Rep*. **6**, 36925; doi: 10.1038/srep36925 (2016).

**Publisher’s note:** Springer Nature remains neutral with regard to jurisdictional claims in published maps and institutional affiliations.

## Figures and Tables

**Table 1:** Clinical characteristics of the participants.

	Men			Women		
	PUD (−)	PUD (+)	*P-value*	PUD (−)	PUD (+)	*P-value*
Numbers	5,670	411		7,805	404	
Age (years)	43.0 ± 0.31	51.9 ± 0.75	<0.001	45.3 ± 0.32	53.6 ± 0.87	<0.001
Body mass index (kg/m^2^)	24.1 ± 0.05	23.4 ± 0.17	<0.001	23.2 ± 0.06	23.7 ± 0.21	0.024
Waist circumference (cm)	84.1 ± 0.18	83.7 ± 0.54	0.573	78.0 ± 0.20	80.4 ± 0.62	0.001
Smoker, current (%)	47.4 (0.77)	47.9 (2.85)	0.866	7.0 (0.38)	6.9 (1.54)	0.969
Drinking, heavy (%)	32.5 (0.71)	35.7 (3.25)	0.319	5.8 (0.32)	8.3 (1.81)	0.090
Regular exercise (%)	28.1 (0.79)	28.1 (2.63)	0.990	23.6 (0.74)	26.7 (2.66)	0.239
Education, ≥high school (%)	77.3 (0.75)	62.1 (2.66)	<0.001	64.0 (0.89)	45.0 (3.10)	<0.001
Income, lowest quartile (%)	14.4 (0.67)	16.7 (2.00)	0.253	17.3 (0.70)	27.1 (2.31)	<0.001
Spouse, yes (%)	69.1 (1.02)	87.1 (2.15)	<0.001	65.6 (0.79)	66.3 (2.71)	0.810
Occupation yes (%)	76.3 (0.81)	77.0 (2.60)	0.802	47.3 (0.75)	43.7 (2.78)	0.209
Diabetes mellitus (%)	8.6 (0.42)	12.9 (1.88)	0.010	7.7 (0.37)	7.9 (1.53)	0.865
Hypertension (%)	29.1 (0.79)	32.9 (2.71)	0.166	22.7 (0.65)	31.9 (2.46)	<0.001
Stress (%)	27.4 (0.64)	34.9 (2.89)	0.007	31.6 (0.65)	41.4 (3.01)	0.001
Depressive symptoms (%)	10.0 (0.44)	15.4 (2.04)	0.002	19.1 (0.59)	29.9 (2.63)	<0.001
Suicidal ideation (%)	10.8 (0.49)	15.9 (2.20)	0.008	22.0 (0.63)	30.5 (2.70)	0.001
Sleep mean duration (hours)	6.9 ± 0.02	6.7 ± 0.08	0.014	6.9 ± 0.02	6.5 ± 0.09	<0.001
Sleep duration (%)			0.169			<0.001
<5 hours	11.9 (0.46)	13.9 (1.81)		15.1 (0.47)	23.1 (2.50)	
6 hours	28.9 (0.66)	32.7 (2.84)		23.8 (0.57)	24.5 (2.46)	
7 hours	29.4 (0.68)	29.7 (2.73)		28.6 (0.63)	25.7 (2.65)	
8 hours	22.5 (0.61)	17.2 (1.97)		22.8 (0.54)	21.3 (2.28)	
≥9 hours	7.3 (0.42)	6.4 (1.45)		9.7 (0.39)	5.4 (1.12)	

Data are presented as the mean ± SE, or percentage (SE).

PUD, peptic ulcer disease.

**Table 2:** Characteristics of subjects across categories of sleep duration.

	Men (N = 6,081)						Women (N = 8,209)					
	<5 hours	6 hours	7 hours	8 hours	≥9 hours	*P*	<5 hours	6 hours	7 hours	8 hours	≥9 hours	*P*
Number	826	1,693	1,718	1,348	496		1,395	1,889	2,329	1,841	755	
Peptic ulcer disease (%)	6.5 (0.87)	6.3 (0.65)	5.7 (0.62)	4.4 (0.56)	5.0 (1.13)	0.169	5.96 (0.72)	4.1 (0.49)	3.6 (0.42)	3.7 (0.44)	2.2 (0.46)	<0.001
Age (years)	48.8 (0.75)	42.8 (0.42)	42.6 (0.41)	42.7 (0.50)	44.3 (1.06)	<0.001	55.1 (0.60)	45.9 (0.44)	43.7 (0.42)	42.6 (0.49)	43.1 (0.83)	<0.001
Body mass index (kg/m2)	24.2 (0.14)	24.1 (0.09)	24.1 (0.08)	23.9 (0.10)	23.5 (0.21)	0.021	23.9 (0.12)	23.3 (0.09)	23.1 (0.09)	22.9 (0.10)	22.9 (0.16)	<0.001
Waist circumference (cm)	85.1 (0.38)	84.2 (0.28)	83.9 (0.26)	83.6 (0.30)	83.1 (0.58)	0.010	80.8 (0.37)	78.1 (0.28)	77.3 (0.29)	77.4 (0.31)	77.5 (0.44)	<0.001
Smoker, current (%)	46.5 (2.00)	46.4 (1.42)	46.2 (1.43)	50.2 (1.40)	49.1 (2.67)	0.267	8.3 (0.93)	6.3 (0.68)	5.4 (0.54)	7.7 (0.75)	10.0 (1.26)	<0.001
Drinking, heavy (%)	37.4 (1.96)	32.0 (1.25)	34.2 (1.32)	30.7 (1.55)	27.9 (2.44)	0.014	6.2 (0.76)	6.0 (0.67)	4.5 (0.47)	6.8 (0.80)	7.0 (1.11)	0.061
Regular exercise (%)	24.6 (1.76)	29.6 (1.29)	29.7 (1.36)	27.1 (1.42)	23.6 (2.35)	0.028	23.7 (1.43)	25.3 (1.35)	25.2 (1.16)	23.2 (1.14)	16.8 (1.49)	<0.001
Education, ≥high school (%)	62.3 (2.01)	80.2 (1.12)	79.9 (1.12)	77.3 (1.29)	67.9 (2.47)	<0.001	39.2 (1.81)	62.2 (1.36)	70.5 (1.17)	71.2 (1.44)	63.7 (2.02)	<0.001
Income, lowest quartile (%)	20.7 (1.58)	11.7 (0.93)	11.9 (0.94)	15.6 (1.17)	23.7 (2.05)	<0.001	31.5 (1.61)	15.6 (0.99)	14.1 (0.88)	14.1 (0.94)	20.0 (1.73)	<0.001
Spouse, yes (%)	73.1 (1.88)	71.3 (1.54)	73.0 (1.41)	66.9 (1.68)	58.3 (3.07)	<0.001	58.3 (1.65)	66.5 (1.35)	69.5 (1.16)	67.1 (1.37)	60.6 (2.10)	<0.001
Occupation, yes (%)	71.5 (1.80)	82.1 (1.23)	79.7 (1.20)	72.9 (1.41)	57.9 (2.81)	<0.001	43.1 (1.50)	52.5 (1.35)	51.3 (1.27)	45.8 (1.40)	31.1 (2.08)	<0.001
Diabetes mellitus (%)	10.5 (1.18)	8.5 (0.78)	7.9 (0.71)	8.8 (0.79)	11.1 (1.79)	0.193	12.9 (0.98)	6.7 (0.61)	6.4 (0.59)	6.7 (0.63)	8.0 (1.15)	<0.001
Hypertension (%)	32.8 (1.90)	28.2 (1.24)	28.6 (1.27)	29.4 (1.39)	30.4 (2.36)	0.265	37.8 (1.61)	21.5 (1.03)	20.5 (1.07)	18.1 (1.15)	23.0 (1.82)	<0.001
Stress (%)	36.5 (1.88)	30.2 (1.26)	27.8 (1.24)	22.1 (1.32)	21.7 (2.17)	<0.001	40.6 (1.60)	33.5 (1.26)	29.9 (1.05)	28.2 (1.24)	30.3 (1.98)	<0.001
Depressive symptoms (%)	17.0 (1.38)	9.3 (0.86)	8.4 (0.75)	9.4 (0.96)	13.4 (1.87)	<0.001	27.3 (1.38)	19.4 (1.07)	16.5 (0.95)	18.1 (1.11)	20.3 (1.78)	<0.001
Suicidal ideation (%)	18.9 (1.59)	10.7 (0.80)	7.9 (0.74)	10.4 (1.07)	15.4 (2.07)	<0.001	31.7 (1.57)	21.4 (1.10)	18.3 (0.94)	20.3 (1.06)	26.2 (1.90)	<0.001

Data are presented as the mean ± SE, or percentage (SE).

**Table 3:** Adjusted odds ratios for the prevalence of peptic ulcer disease according to sleep duration.

		<5 hours	6 hours	7 hours	8 hours	≥9 hours
		OR (95% CI)	OR (95% CI)	OR (95% CI)	OR (95% CI)	OR (95% CI)
Men	Model 1	0.90 (0.628–1.285)	1.13 (0.828–1.541)	1	0.74 (0.530–1.041)	0.74 (0.443–1.223)
	Model 2	0.92 (0.638–1.319)	1.16 (0.849–1.576)	1	0.77 (0.551–1.087)	0.77 (0.453–1.300)
	Model 3	0.85 (0.584–1.233)	1.14 (0.835–1.559)	1	0.79 (0.561–1.103)	0.76 (0.447–1.277)
Women	Model 1	1.21 (0.830–1.750)	1.06 (0.741–1.524)	1	1.06 (0.764–1.483)	0.59 (0.363–0.950)
	Model 2	1.20 (0.823–1.753)	1.20 (0.823–1.753)	1	1.06 (0.758–1.473)	0.60 (0.369–0.978)
	Model 3	1.12 (0.768–1.632)	1.03 (0.717–1.469)	1	1.06 (0.758–1.476)	0.59 (0.362–0.964)

Model 1: adjusted for age and body mass index.

Model 2: adjusted for age, body mass index, smoking, drinking, exercise, education, income, and spouse.

Model 3: adjusted for age, body mass index, smoking, drinking, exercise, education, income, spouse, stress, and depressive symptoms.
